# Use of ^18^F-FDG PET/CT to Differentiate Ectopic Adrenocorticotropic Hormone-Secreting Lung Tumors From Tumor-Like Pulmonary Infections in Patients With Ectopic Cushing Syndrome

**DOI:** 10.3389/fonc.2021.762327

**Published:** 2021-10-08

**Authors:** Guozhu Hou, Yuanyuan Jiang, Fang Li, Xin Cheng

**Affiliations:** ^1^ Department of Nuclear Medicine, Peking Union Medical College Hospital, Chinese Academy of Medical Sciences and Peking Union Medical College, Beijing, China; ^2^ Beijing Key Laboratory of Molecular Targeted Diagnosis and Therapy in Nuclear Medicine, Beijing, China

**Keywords:** ectopic Cushing syndrome, adrenocorticotropic hormone-secreting lung tumors, pulmonary infections, fluorodeoxyglucose, positron emission tomography/computed tomography

## Abstract

**Background:**

Ectopic adrenocorticotropic hormone (ACTH)-secreting lung tumors represent the most common cause of ectopic Cushing syndrome (ECS). Pulmonary opportunistic infections are associated with ECS. The present study aimed to evaluate the usefulness of ^18^F-FDG PET/CT for differentiating ectopic ACTH-secreting lung tumors from tumor-like pulmonary infections in patients with ECS.

**Methods:**

We retrospectively reviewed the imaging data of 24 patients with ECS who were suspected to have ACTH-secreting lung tumors and underwent ^18^F-FDG PET/CT between 2008 and 2019. Eleven patients with lung tumors and 4 with pulmonary infections also had additional somatostatin receptor imaging (^99m^Tc-HYNIC-TOC SPECT/CT or ^68^Ga-DOTATATE PET/CT).

**Results:**

In total, 18 patients had lung tumors and six had pulmonary infections. The primary source of ECS remained occult in the six patients with pulmonary infections. The maximum standardized uptake value (SUV_max_) for pulmonary infections was significantly higher than that for tumors (*P* = 0.008). Receiver operating characteristic analysis revealed that a cut-off SUV_max_ of 4.95 helped in differentiating ACTH-secreting lung tumors from infections with 75% sensitivity and 94.4% specificity. For the 11 patients with ACTH-lung tumors, somatostatin receptor imaging (SRI) was positive in 6; while for the 4 with pulmonary infections, SRI was positive in 2. The sensitivity and specificity of somatostatin receptor imaging (SRI) for detecting ACTH-secreting lung tumor was 54.5% and 50%.

**Conclusions:**

Our findings suggest that pulmonary infections exhibit significantly higher FDG uptake than ACTH-secreting lung tumors in ^18^F-FDG PET/CT. An SUV_max_ cut-off value of 4.95 may be useful for differentiating the two conditions. Our results also suggested that SRI may not be an effective tool for differentiating the two conditions given the relatively low specificity.

## Introduction

10%-15% of Cushing syndrome is caused by ectopic adrenocorticotropic hormone (ACTH)-secreting tumors. In such cases, resection of the tumors can have curative effects. The most common tumors associated with ECS are pulmonary carcinoids and small cell lung carcinoma (SCLC), followed by thymic carcinoids, pancreatic neuroendocrine tumors, medullary thyroid carcinoma, and pheochromocytoma (1). ^18^F-fluorodeoxyglucose (FDG) positron emission tomography (PET)/computed tomography (CT; ^18^F-FDG PET/CT) has been shown to be an effective modality for localizing ectopic ACTH-secreting tumors causing ECS. Pulmonary carcinoids generally demonstrate low to moderate metabolic activity because of their low proliferation rate and slow growth. Meanwhile, ACTH-producing SCLC can show positive findings on ^18^F-FDG PET/CT, although the reported number of ECS-causing SCLCs detected by ^18^F-FDG PET/CT is quite small. This is probably because the patients are rapidly diagnosed by conventional cross-sectional imaging and do not undergo further ^18^F-FDG PET/CT (2).

ECS due to ectopic ACTH-secreting tumors is associated with markedly elevated ACTH levels. This results in high circulating glucocorticoid levels, which could affect cell-mediated immunity (3) and impair immune function by inhibiting the phagocytic function of alveolar macrophages and reducing neutrophil recruitment to the infected areas. This results in an increased incidence of opportunistic bacterial and fungal infections (4, 5). The four most common infections associated with ECS are cryptococcosis, aspergillosis, nocardiosis, and pneumocystosis (6), with the lung being the most frequently involved site. Pulmonary infections can exhibit varied radiographic findings and may appear as nodules or masses simulating lung tumors (7). Thus, it could be difficult for conventional anatomic imaging to differentiate tumor-like pulmonary infections from lung tumors. FDG is a nonspecific tracer that accumulates in areas of infection. Pulmonary cryptococcosis, aspergillosis, nocardiosis, and pneumocystosis have been reported to show high metabolic activity and mimic lung malignancies on ^18^F-FDG PET/CT (8–11). In the clinical setting of immunosuppression resulting from ECS, surgery for the removal of pulmonary infectious lesions can deteriorate the patient’s condition. Therefore, discrimination of infections and tumors is crucial for avoiding unnecessary surgical intervention. The primary goal of this retrospective study was to evaluate the usefulness of ^18^F-FDG PET/CT for differentiating ectopic ACTH-secreting lung tumors from tumor-like pulmonary infections in patients with ECS. In addition, as somatostatin receptor-based imaging (SRI) has been increasingly used for the detection of occult ECS tumors, we also reviewed the SRI imaging findings in 15 patients and investigated whether the SRI findings were able to differentiate the two conditions.

## Materials and Methods

### Patients

We retrospectively reviewed ^18^F-FDG PET/CT scans obtained for localizing the source of ectopic ACTH secretion in all patients with ECS in our department between 2008 and 2019. The inclusion criteria were as follows: (a) confirmed diagnosis of ACTH-dependant Cushing syndrome; (b) negative finding on pituitary MRI; (c) lung nodules suspected on chest CT images. Exclusion criteria were as follows: (a) confirmed tumoral source other than in the lung (i.e. thymus, gastrointestinal tract and pancreatic neuroendocrine tumor), (b) unavailable histopathological result of suspected lung lesion. Eventually, 24 patients with suspicious ACTH-secreting lung tumors were included in the present study. The diagnosis of ECS was confirmed by clinical presentations combined with laboratory tests including low-dose dexamethasone suppression test (LDDST), high-dose dexamethasone suppression test (HDDST), CRH test, inferior petrosal sinus sampling (IPSS). The head MRI results of all patients suggested that the pituitary gland was normal. Pulmonary CT indicates pulmonary nodules, but the nature is unclear. Fifteen patients also underwent additional ^99m^Tc-HYNIC SPECT/CT (n=6) or ^68^Ga-DOTATATE PET/CT (n=9). The reference standard was histopathological diagnosis obtained by either lung surgery or biopsy. There were 11 female and 13 male patients aged 9 to 72 years (mean age, 37.8 ± 17.1 years). This retrospective study of existing patient data and images was approved by the institutional review board of Peking Union Medical College Hospital. The requirement for informed consent was waived.

### ^18^F-FDG PET/CT Study

Following 8 h of fasting and confirming the blood glucose level to be less than 120 mg/dL, ^18^F-FDG (5.5 MBq/kg) was intravenously injected. An hour later, PET/CT images were acquired from the mid-thigh to the skull base (2 min/bed position) using a combined PET/CT Biograph (Siemens Co.). All scans were obtained in a three-dimensional model.

### ^99m^Tc-HYNIC-TOC Scintigraphy

^99m^Tc-HYNIC-TOC was synthesized and labeled as previously described (12). After intravenous administration of the tracer, whole-body planar images were acquired using a double-head gamma camera at 1 and 4 hours after injection. Some patients also underwent pulmonary SPECT/CT imaging when there is an increased uptake in the chest.

### ^68^Ga-DOTA-TATE PET/CT Study

The ^68^Ga-DOTATATE was produced following our previously published procedure (13). The study was carried out on a PET/CT scanner (Siemens Co.). Patients received an intravenous injection of ^68^Ga-DOTATATE (111-148 MBq). A low-dose whole-body CT scan was obtained at 40-60 min post-injection for anatomical localization and attenuation correction. PET scanning followed at 1.5 min/bed position with a 23-slice overlap. Images were reconstructed using an ordered subsets expectation-maximization algorithm and corrected for CT-based attenuation, dead time, random events, and scatter.

### Image Interpretation and Statistical Analysis

The images were reviewed by two experienced nuclear medicine physicians, who visually inspected the images and performed semi-quantitative measurements based on the maximum standard uptake value (SUV_max_), which is determined by selecting the point of maximum FDG uptake within the lesion. For ^99m^Tc-HYNIC SPECT/CT and ^68^Ga-DOTATATE PET/CT, the images were interpreted as positive if the tracer uptake in the lesion was higher than surrounding background.

All data are expressed as mean ± standard deviation. Differences between groups were analyzed using the Student *t* test, nonparametric analysis, and χ2 test. The cut-off SUV_max_ for differentiating pulmonary infections from ACTH-secreting tumors was obtained *via* receiver operating characteristic (ROC) analysis with calculation of areas under the curve (AUCs) and sensitivity and specificity values. The correlation between ACTH level and SUVmax value was evaluated using the Pearson correlation coefficient. A *P*-value of <0.05 was considered statistically significant. All statistical analyses were performed using SPSS Statistics (version 21.0, IBM SPSS Inc., IBM, Chicago, IL, USA).

## Results

### Patients

Among the 24 patients, 18 patients with 18 lesions were diagnosed with ectopic ACTH-secreting tumours (typical carcinoids, n =12; atypical carcinoids, n = 5; SCLC, n =1) while six patients with eight lesions were diagnosed with pulmonary infections (cryptococcosis, n = 3; aspergillosis, n = 4; pulmonary abscess, n = 1). Therefore, a total of 26 lesions were analyzed in this study. The patient characteristics are shown in **Table 1**. The clinical presentations are summarized in **Table 2**. After surgical resection of the lesions, all patients in the tumor group were relieved of all symptoms, with serum cortisol and ACTH levels returning to normal. On the other hand, the source of ectopic ACTH secretion remained occult in patients with pulmonary infections. One patient of the infection group died of cryptococcal meningitis after surgery resection of pulmonary nodule. Thirteen of the tumor group (4 typical carcinoids, 9 typical carcinoids) had follow up information. Two patients with atypical carcinoids developed recurrence, while no patient died during the follow-up period,

**Table 1:** Clinical features of ectopic Cushing syndrome patients, including histopathological characteristics, metastases, size, and SUV_max_ of lesions.

Patient	Sex/age	ACTH (pg/ml)^*^	Histopathological characteristics	Metastases	Diameter (mm)	SUV_max_ (18F-FDG PET/CT)	SRI result
**Infectious lesions**							
**1**	F/60	326	cryptococcus	N/A	7	1.2	Negative
**2**	F/41	59.1	Abscess	N/A	32	6.2	Positive
**3**	F/34	49.5	cryptococcus	N/A	14	5.7	Positive
**3**	F/34	49.5	cryptococcus	N/A	35	12.4	
**4**	M/39	48.5	Aspergillus	N/A	23	1.0	Negative
**5**	M/53	1041	Aspergillus	N/A	32	9.7	NA
**5**	M/53	1041	Aspergillus	N/A	12	5.2	NA
**6**	F/47	N/A	Aspergillus	N/A	12	5.8	NA
**ACTH-secreting tumors**					
**7**	M/28	191	TC (ACTH, +; Ki-67, 1%; TTF-1, +)	**-**	10	0.6	Positive
**8**	M/29	116	TC (ACTH, +; Ki-67, 3%)	**-**	12	4.7	NA
**9**	F/9	115	AC (ACTH, +; Number of mitosis, 1/10 HPF; Ki-67, 15%; TTF-1, -)	**-**	14	1.4	Positive
**10**	M/24	222	TC (ACTH, +; Ki-67, 3%; TTF-1, +)	**+**	17	2.7	Positive
**11**	F/48	153	TC (ACTH, +; Ki-67, 2%)	**-**	5	0.8	NA
**12**	F/27	111	TC (ACTH, +; Ki-67, 3%)	**-**	8	0.9	Negative
**13**	M/22	140	AC (ACTH, +; Number of mitosis, 3/10 HPF; Ki-67, 6%)	**-**	6	0.9	Negative
**14**	M/13	107	TC (ACTH, +; Ki-67, 1%; TTF-1, +)	**+**	10	1.1	Negative
**15**	M/45	68.3	AC (ACTH, +; Number of mitosis, 8/10 HPF; Ki-67, 10%; TTF-1, +)	**+**	10	1.9	Positive
**16**	M/30	100	TC (ACTH, +; Number of mitosis, 1/10 HPF; Ki-67, 10%; TTF-1, +)	**-**	11	0.6	Negative
**17**	F/72	129	TC (ACTH, +; Ki-67, 2%; TTF-1, +)	**-**	15	2.8	NA
**18**	F/44	874	TC (ACTH, +; Ki-67, 1%)	**+**	9	0.7	NA
**19**	F/45	60.6	TC (ACTH, +; Ki-67, 2%; TTF-1, +)	**+**	16	3.8	NA
**20**	F/52	572	TC (ACTH, +; Ki-67, 2%; TTF-1, +)	**+**	14	3.4	Negative
**21**	M/16	130	AC (ACTH, +; Ki-67, 5%; TTF-1, +)	**-**	19	1.1	Positive
**22**	M/12	865	AC (ACTH, +; Ki-67, 2%)	**+**	28	3.0	Positive
**23**	M/62	278	TC (ACTH, +; Ki-67, 1%; TTF-1, +)	**-**	7	0.6	NA
**24**	M/57	261	SCLC (ACTH, +; Ki-67, 25%)	**+**	37	7.7	NA

SUV_max_, maximum standardized uptake value; N/A, not applicable; AC, atypical carcinoid; TC, typical carcinoid; F, female; M, male; SCLC, small cell lung cancer; SRI, somatostatin receptor imaging. ^*^Reference range for ACTH: 0-46 pg/ml.

**Table 2:** Clinical presentations of patients.

Clinical presentations	Patients, n (%)
**Round face**	21, (87.5%)
**Dorsal fat pad**	19, (79.1%)
**Skin alterations**	18, (75%)
**Weight gain**	16, (66.7%)
**Hypertension**	16, (66.7%)
**Hirsutism**	6, (25%)
**Menstrual irregularities**	3, (12.5)
**Hair loss**	1, (4.1%)

### ^18^F-FDG PET/CT Results

**Figure 1:**
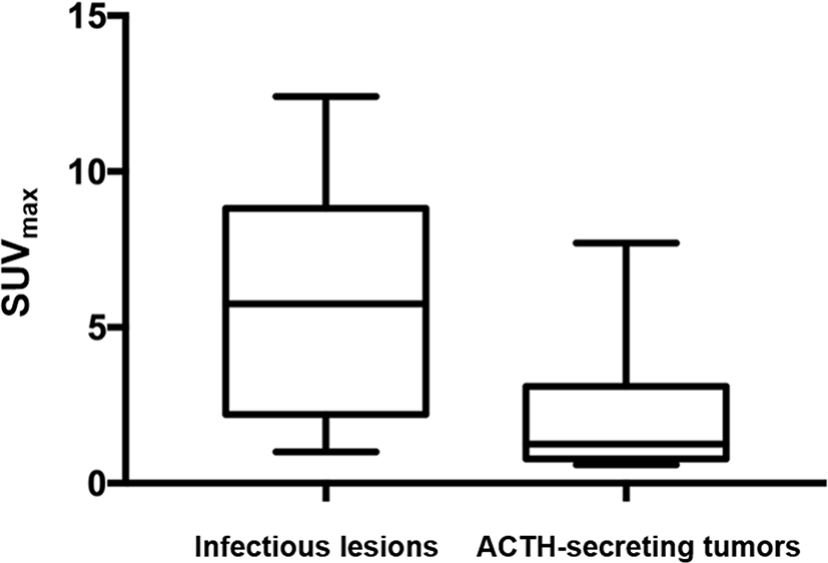
SUV_max_ was significantly higher for infectious lesions than for ACTH-secreting tumors (*P* = 0.008).

**Figure 2:**
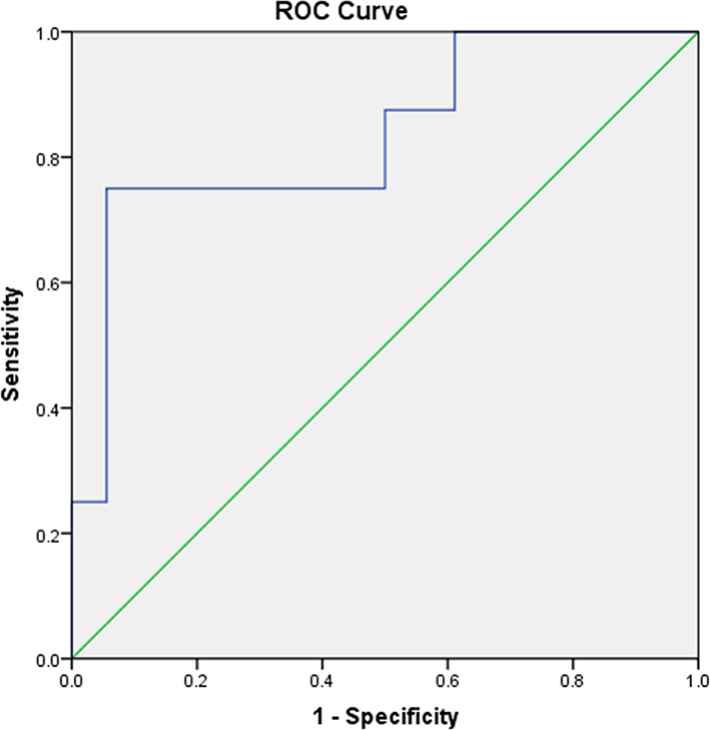
A receiver operating characteristic curve for measuring the accuracy of the SUV_max_ as a parameter for distinguishing pulmonary ACTH-secreting tumors from pulmonary infection. The area under the curve is 0.833. A cut-off SUV_max_ of 4.95 or greater is predictive of pulmonary infection with 75% sensitivity and 94.4% specificity.

**Figure 3:**
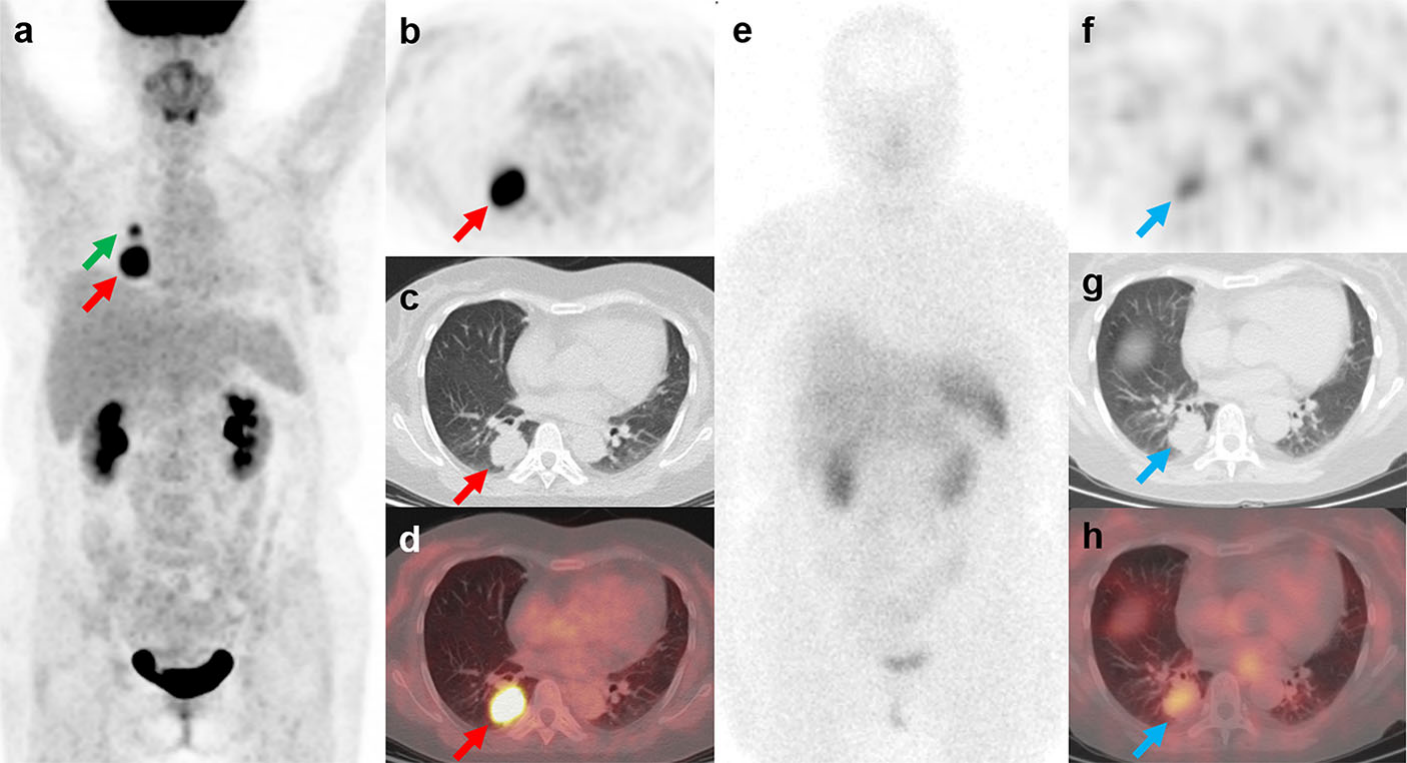
^18^F-FDG PET/CT and ^99m^Tc-HYNIC-TOC SPECT/CT findings in a representative case of cryptococcosis (Patient, 3). A 34-year-old woman with ectopic Cushing syndrome underwent ^18^F-FDG PET/CT and ^99m^Tc-HYNIC-TOC SPECT/CT for the detection of ACTH-secreting tumor. ^18^F-FDG PET/CT showed a mass in the right lung **(A–D**; red arrows, 3.5cm, SUVmax, 12.4). A hypermetabolic nodule near the right hilum was also noted (**A**; green arrow, 1.4cm, SUVmax, 5.7). The mass demonstrated positive uptake on ^99m^Tc-HYNIC-TOC SPECT/CT (**E–H**; blue arrows), and the nodule showed slightly increased uptake. The two lesions were then surgically removed and histopathological results confirmed pulmonary cryptococcosis. Three weeks after surgery, the patient developed severe headache and fever symptoms. Cryptococcus cerebrospinal culture was positive, suggesting cryptococcal meningitis. The patient responded poorly to the antibiotic therapy and died of cerebral hernia.

**Figure 4:**
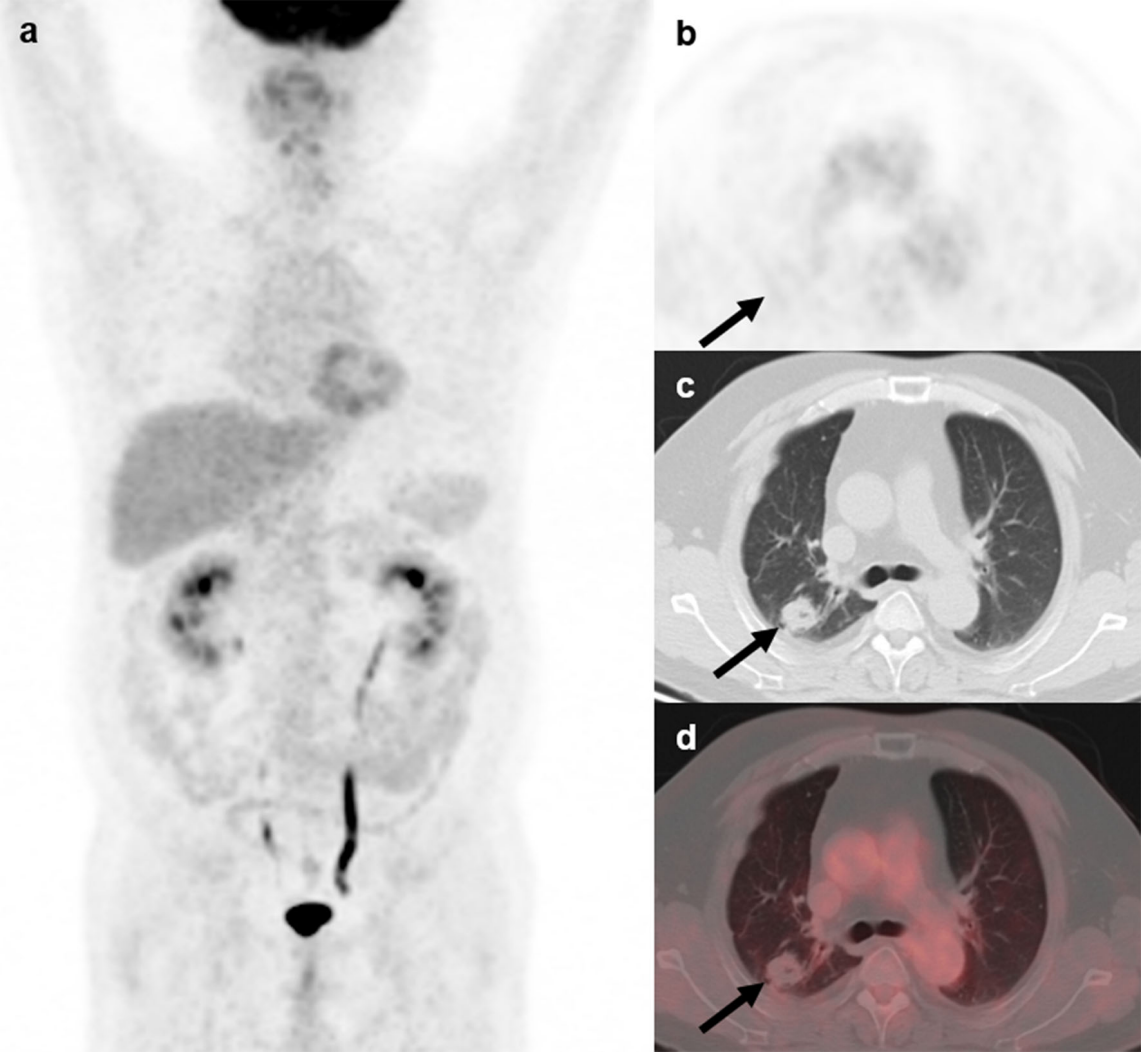
^18^F-FDG PET/CT findings in a representative case of aspergillosis (Patient, 4). A 39-year-old man diagnosed with ectopic Cushing syndrome underwent ^18^F-FDG PET/CT to locate the possible ACTH-secreting tumor. ^18^F-FDG PET/CT **(A)** showed a nodule in the right lung demonstrating slightly higher-than-background activity (**B–D**; arrows; SUV_max_, 1.0). Subsequent percutaneous lung biopsy confirmed the diagnosis of pulmonary aspergilloma.

**Figure 5:**
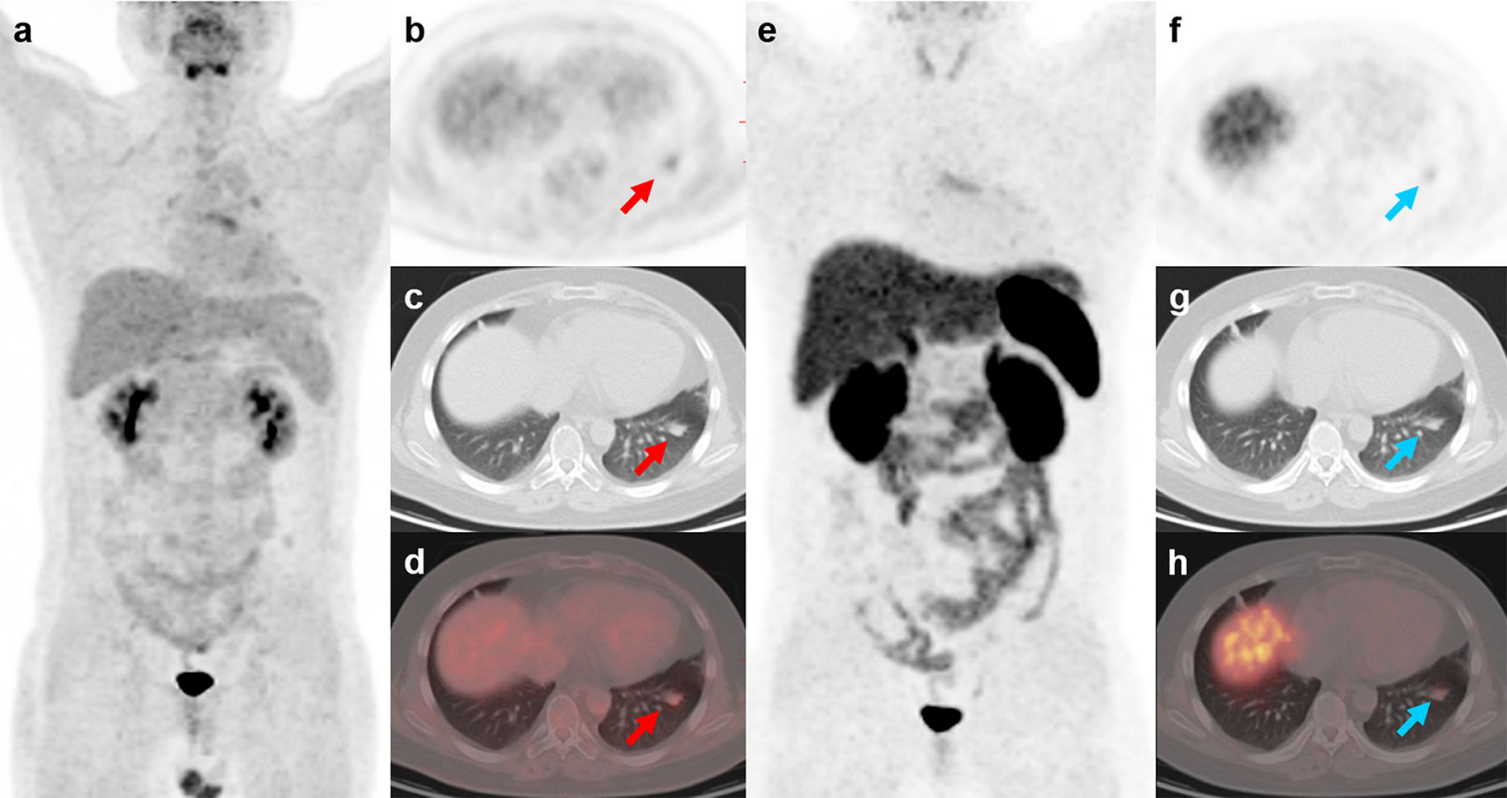
^18^F-FDG and ^68^Ga-DOTATATE PET/CT findings in a representative case of typical carcinoid (Patient, 10). A 24-year-old man with ectopic Cushing syndrome underwent ^18^F-FDG and ^68^Ga-DOTATATE PET/CT for detecting the source tumor of ACTH secretion. ^18^F-FDG PET/CT showed a nodule with mild activity (**A–D**; red arrows; SUVmax, 2.7) in the left lung. ^68^Ga-DOTATATE PET/CT also demonstrated slightly increased uptake in the nodule (**E–H**; blue arrows; SUVmax, 2.0), indicating somatostatin receptor expression. The nodule was then surgically removed and postoperative histopathology confirmed typical carcinoid with ACTH positivity in immunohistochemistry.

**Figure 6:**
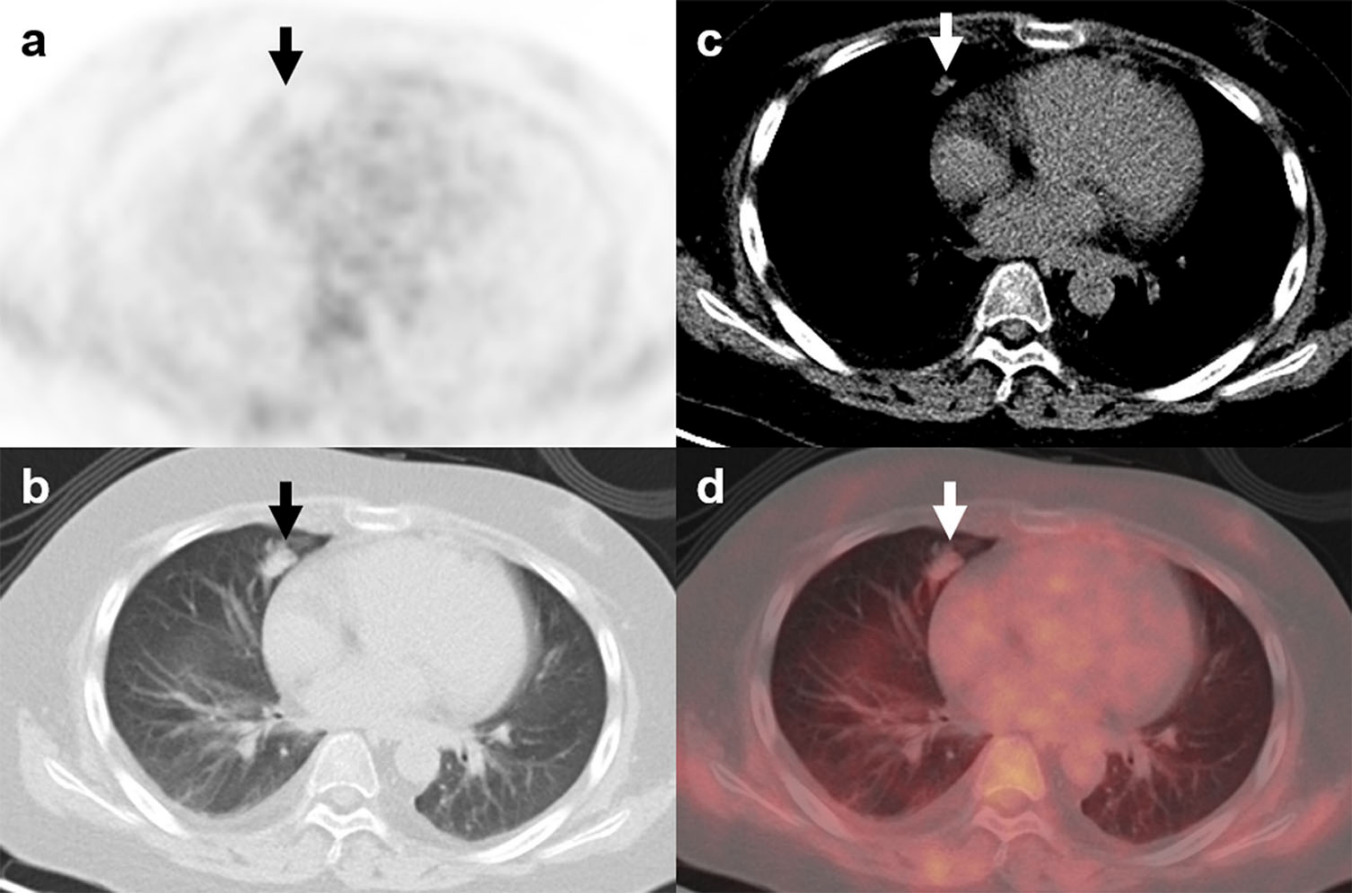
^18^F-FDG PET/CT findings in a representative case of atypical carcinoid (Patient, 21). A 16-year-old man was diagnosed with ectopic Cushing syndrome. The patient underwent ^18^F-FDG PET/CT to localize the source of ACTH secretion. PET/CT images revealed a nodule with mild FDG activity (**A–D**; arrows; SUV_max_, 1.1) in the right lung. The lesion was surgically removed, and histopathological analysis confirmed the diagnosis of atypical carcinoid with ACTH positivity in immunohistochemistry.

**Figure 7:**
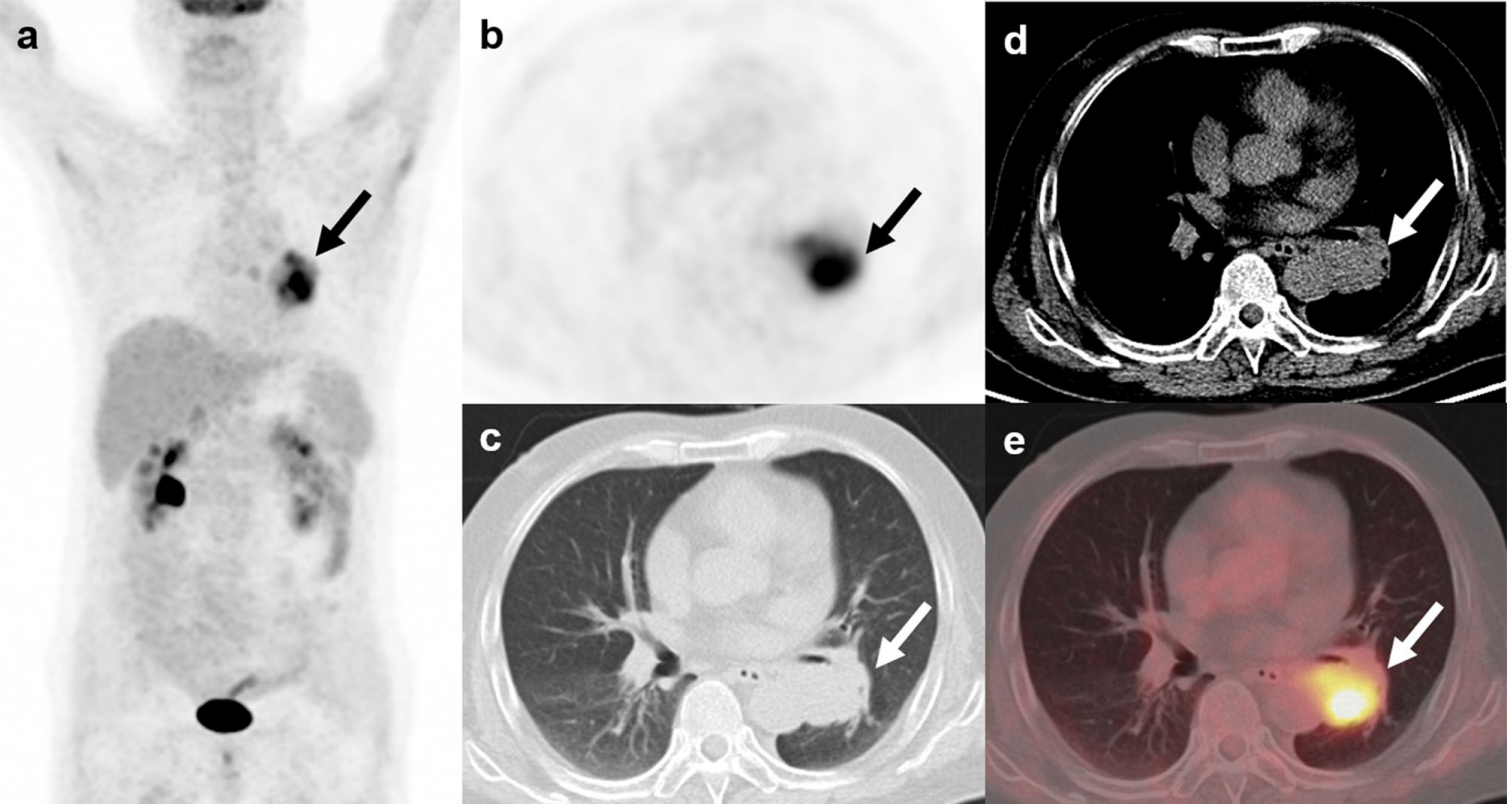
^18^F-FDG PET/CT findings in a representative case of small cell lung cancer (Patient, 24). A 57-year-old man diagnosed with ectopic Cushing syndrome underwent ^18^F-FDG PET/CT to localize the source of ACTH secretion. PET/CT images demonstrate a highly FDG-avid mass (**A–E**; arrows; SUV_max_, 7.7) adjacent to the left hilum. The mass was then surgically removed and postoperative histopathology confirmed small cell lung cancer with ACTH positivity in immunohistochemistry.

The mean SUV_max_ for all 18 lesions in the patients with ectopic ACTH-secreting lung tumors was 2.1 ± 1.8 (range: 0.6–7.7), while that for the eight lesions in the patients with pulmonary infections was 5.9 ± 3.8 (range: 1.0–12.4). Thus, SUV_max_ was significantly higher for infectious lesions than for tumors (*P* = 0.008; **Figure 1**). ROC curve analysis suggested that an SUV_max_ of ≥4.95 was predictive of pulmonary infection with 75% sensitivity and 94.4% specificity; AUC was 0.833 (standard error, 0.093; *P* = 0.008; 95% confidence interval, 0.651–1.000; **Figure 2**). The mean diameters of the ectopic ACTH-secreting lung tumors and pulmonary infectious lesions were 13.8 ± 7.9 (range: 5–37) and 20.9 ± 11.0 (range: 7–35) mm, respectively, with no significant between-group difference (*P* = 0.126; **Table 3**). **Figures 3**–**7** present representative cases of cryptococcosis (two lesions; SUV_max_, 5.7 and 12.4), aspergillosis (SUV_max_, 1.0), a typical carcinoid (SUV_max_, 2.7), an atypical carcinoid (SUV_max_, 1.1), and small cell lung cancer (SUV_max_, 7.7), respectively. The ACTH level was not significantly correlated with the SUVmax value for ACTH-secreting lung tumors (P=0.816).

**Table 3:** Imaging characteristics of ectopic Cushing syndrome patients.

	ACTH-secreting tumors (n = 18)	Infectious lesions (n = 8)	*P* value
**Diameter (mm)**	13.8 ± 7.9 (5-37)	20.9 ± 11.0 (7-35)	0.126
**SUV_max_**	2.1 ± 1.8 (0.6-7.7)	5.9 ± 3.8 (1.0-12.4)	0.008

SUV_max_, maximum standardized uptake value.

### SRI Results

Four patients of the infection group had SRI, of which 3 had ^99m^Tc-HYNIC SPECT/CT and 1 had ^68^Ga-DOTATATE PET/CT. Two of the 4 patients had positive imaging results. Eleven patients of the tumor group had SRI, of which 3 had ^99m^Tc-HYNIC SPECT/CT and 8 had ^68^Ga-DOTATATE PET/CT. Six of the 11 patients had positive imaging results. Therefore, the sensitivity and specificity of SRI was 54.5% and 50%, respectively.

## Discussion

Ectopic ACTH-producing tumors account for 15%–20% of cases of ACTH-dependent Cushing syndrome. Lung carcinoids and SCLC represent the most common tumors associated with ECS, and the resection of the responsible tumors can have curative effects (14). There is no consensus regarding the usefulness of ^18^F-FDG PET/CT for localizing the source of ectopic ACTH secretion, even though it is the most commonly used molecular imaging method in clinical practice. A nodule or mass-like lesion in the lung that demonstrates abnormal activity on ^18^F-FDG PET/CT tends to be interpreted as an ACTH-secreting tumor and might be surgically resected. However, in clinical practice, the resected pulmonary ‘tumor’ occasionally turns out to be an infectious lesion most often caused by fungus. In such cases, surgery is unnecessary and may deteriorate the patient’s condition. The present study included 18 patients with ectopic ACTH-secreting lung tumors and six patients with pulmonary infections. To the best of our knowledge, this is the first study to describe and compare the features of ACTH-secreting lung tumors and pulmonary infectious pseudotumors using ^18^F-FDG PET/CT. This discrimination is important because the two conditions require different treatment plans.

We found that a cut-off SUV_max_ of 4.95 maximized the sensitivity and specificity for the differentiation of pulmonary infections from ACTH-secreting tumors. Specifically, the findings indicated that a pulmonary nodule or mass-like lesion with a SUV_max_ of ≥4.95 was more likely to be an infectious lesion. Our study included only one SCLC, and it was the only lesion with a SUV_max_ of >4.95 in the tumor group (SUV_max_, 7.7). SCLCs generally exhibit high FDG uptake on PET/CT because of their aggressiveness and high metabolic activity (15). The SCLC was underrepresented in our series, probably because most SCLCs are rapidly diagnosed by conventional cross-sectional imaging and do not require ^18^F-FDG PET/CT or other nuclear imaging modalities for localization (2).

The present study showed significantly higher FDG accumulation in infectious lesions than in pulmonary carcinoids. The reason for the low FDG uptake of pulmonary carcinoids is that most of the lesions (17/18) are well-differentiated neuroendocrine neoplasms (16). As mentioned above, patients with poorly differentiated neuroendocrine neoplasms such as SCLC are rarely examined by FDG PET/CT, which is why we have fewer patients in this group. Among the eight infectious lesions, only two showed low FDG uptake with a SUV_max_ of <4.95. One of the lesions (**Patient 1**, SUV_max_, 1.2) was due to cryptococcosis, and it was the smallest lesion among the infectious lesions (0.7 cm in diameter). The other infectious lesion with low FDG uptake was an aspergilloma (**Patient 4**, SUV_max_, 1.0). Pulmonary aspergillosis can be divided into four subtypes on the basis of clinical and radiological findings: aspergilloma, allergic bronchopulmonary aspergillosis, chronic necrotizing aspergillosis, and invasive pulmonary aspergillosis (IPA) (17). The first three subtypes are also considered to be non-invasive pulmonary aspergillosis (NIPA) (17). Kim et al. evaluated the FDG PET/CT scans of 24 patients with pulmonary aspergillosis (8 IPA and 16 NIPA) and concluded that an isometabolic pattern on FDG PET/CT most likely represented NIPA (18). NIPA is a chronic infection with low virulence and a mild inflammatory reaction, which might attribute to the low metabolic activity on ^18^F-FDG PET/CT.

Somatostatin receptor-based imaging techniques including octreoscan-SPECT/CT and 68Ga-DOTATATE PET/CT have also been shown to be helpful in localizing the source of ECS (19). ^68^Ga-SSTR PET/CT demonstrated high sensitivity in detecting ECS tumors. Two recent systematic reviews reported a sensitivity of 76.1% and 81.8% for ^68^Ga-SSTR PET/CT (2, 20). However, in this study, the sensitivity (54.5%) of SRI (^68^Ga-DOTATATE PET/CT and ^99m^Tc-HYNIC-SPECT/CT) was lower than previously reported. One reason may be publication bias as negative results tend to be not reported. Another reason might be that our study only included patients with lung tumors. Better performance of ^68^Ga-SSTR PET/CT is reported in ACTH-secreting gastroenterpancreatic (GEP) neuroendocrine tumor (NET) (2, 21). Goroshi et al. reported similar ^68^Ga-DOTANOC sensitivity of 60% (6/10) to ours for lung lesions while 100% (3/3) for GEP-NETs (21). Our results also showed that the specificity of SRI (50%) is quite low as two cases of the infection group showed positive finding on ^99m^Tc-HYNIC-TOC SPECT/CT, which were misdiagnosed as the culprit tumors of ECS and were then surgically resected. Inflammatory disease demonstrating increased tracer uptake on SRI is not uncommon in the literature because somatostatin receptor is known to be expressed on activated macrophages in inflammatory process (22, 23). These findings suggested that SRI may not effectively discriminate tumor-like infectious lesions from ACTH-secreting lung tumors.

The main limitations of this study were the small sample size, which does not allow for powerful statistical analysis, and retrospective design. In addition, the number of patients who had follow-up data is small, thus we did not perform statistical analysis on survival. The prognostic value of ^18^F-FDG PET/CT needs to be investigated in future study. Finally, as 6 of the 15 patients who had SRI had ^99m^Tc-HYNIC-TOC SPECT/CT, we could not perform semi-quantitative analysis. Future studies are required to investigate whether there is a difference in intensity of ^68^Ga-DOTATATE uptake between lung infectious lesions and tumors.

In conclusion, although pulmonary infectious lesions associated with ECS and ACTH-secreting lung tumors might exhibit similar morphological features, the former may show higher FDG activity on ^18^F-FDG PET/CT. An SUVmax of 4.95 may help differentiate the two conditions. While our results suggested that SRI may not be an effective tool for differentiating the two conditions given the relatively low specificity.

## Data Availability Statement

The original contributions presented in the study are included in the article/supplementary material. Further inquiries can be directed to the corresponding authors.

## Ethics Statement

Written informed consent was not obtained from the individual(s) for the publication of any potentially identifiable images or data included in this article.

## Author Contributions

GH, YJ, FL, and XC contributed to the design and implementation of the research, to the analysis of the results, and to the writing of the manuscript. All authors contributed to the article and approved the submitted version.

## Funding

This work is supported by National Natural Science Foundation of China (no.81501513), Chinese Academy of Medical Sciences Initiative for Innovative Medicine (CAMS-I2M) 2017-I2M-1-001, and the National Key Research and Development Program of China (No. 2016YFC0901500).

## Conflict of Interest

The authors declare that the research was conducted in the absence of any commercial or financial relationships that could be construed as a potential conflict of interest.

## Publisher’s Note

All claims expressed in this article are solely those of the authors and do not necessarily represent those of their affiliated organizations, or those of the publisher, the editors and the reviewers. Any product that may be evaluated in this article, or claim that may be made by its manufacturer, is not guaranteed or endorsed by the publisher.
